# A tyrosinase, mTyr-CNK, that is functionally available as a monophenol monooxygenase

**DOI:** 10.1038/s41598-017-17635-0

**Published:** 2017-12-08

**Authors:** Hyunsu Do, Eungsu Kang, Byeongseon Yang, Hyung Joon Cha, Yoo Seong Choi

**Affiliations:** 1 Department of Chemical Engineering and Applied Chemistry, Chungnam National University, Daejeon, 34134 South Korea; 2 Department of Chemical Engineering, Pohang University of Science and Technology, Pohang, 37673 South Korea

## Abstract

Tyrosinase efficiently catalyzes the *ortho*-hydroxylation of monophenols and the oxidation of diphenols without any additional cofactors. Although it is of significant interest for the biosynthesis of catechol derivatives, the rapid catechol oxidase activity and inactivation of tyrosinase have hampered its practical utilization as a monophenol monooxygenase. Here, we prepared a functional tyrosinase that exhibited a distinguished monophenolase/diphenolase activity ratio (*V*_max_ mono/ *V*_max_ di = 3.83) and enhanced catalytic efficiency against _L_-tyrosine (*k*_*cat*_ = 3.33 ± 0.18 s^−1^, *K*_*m*_ = 2.12 ± 0.14 mM at 20 °C and pH 6.0). This enzyme was still highly active in ice water (>80%), and its activity was well conserved below 30 °C. *In vitro* DOPA modification, with a remarkably high yield as a monophenol monooxygenase, was achieved by the enzyme taking advantage of these biocatalytic properties. These results demonstrate the strong potential for this enzyme’s use as a monophenol monooxygenase in biomedical and industrial applications.

## Introduction

Tyrosinases, as type-3 (dinuclear) copper proteins, catalyze the oxidation of monophenols (monophenolase activity) and diphenols (diphenolase activity) in the presence of oxygen without any additional co-factors. Tyrosinases are ubiquitously distributed in bacteria and eukaryotes and are mainly involved in the initial stages of melanin biosynthesis for protection against UV and ionizing radiation; the browning of hairs, fruits and vegetables; and other important biological functions^1–4^. In particular, the ability of tyrosinases, unlike other type-3 copper proteins, to *ortho*-hydroxylate monophenols has attracted large amounts of attention for its possible application to the biosynthesis of catechol derivatives, including 3,4-dihydroxyphenylalanine (DOPA), phenolic phytochemicals and DOPA/catechol-tethered polymers and other versatile chemical compounds^2,5–7^. The manufacture of catechol derivatives by chemical routes is relatively difficult due to low yields and the employment of aggressive reagents, complicated starting materials, and multiple reaction steps^8,9^. A high monophenolase/diphenolase activity ratio and/or effective inhibition of diphenolase activity is preferred;^5,10^ however, a review of the *k*_*cat*_ and *K*_*m*_ values of known tyrosinases in the BRENDA server (a comprehensive enzyme information database; http://www.brenda-enzymes.org) shows that the monophenolase activity of most known tyrosinases is much lower than their diphenolase activity. Moreover, catechol groups easily oxidize to become quinones; as a representative example, DOPA is unstable at elevated pH values and high temperatures, undergoing facile oxidation to dopaquinone^7,11^. It is advantageous when the biocatalytic processes are conducted under reaction conditions that minimize autooxidation of the catechol groups. Moreover, enhanced tyrosinases with high catalytic efficiency and stability under these reaction conditions could be a prerequisite for their suitability for biotechnological applications^12,13^.

**Figure 1:**
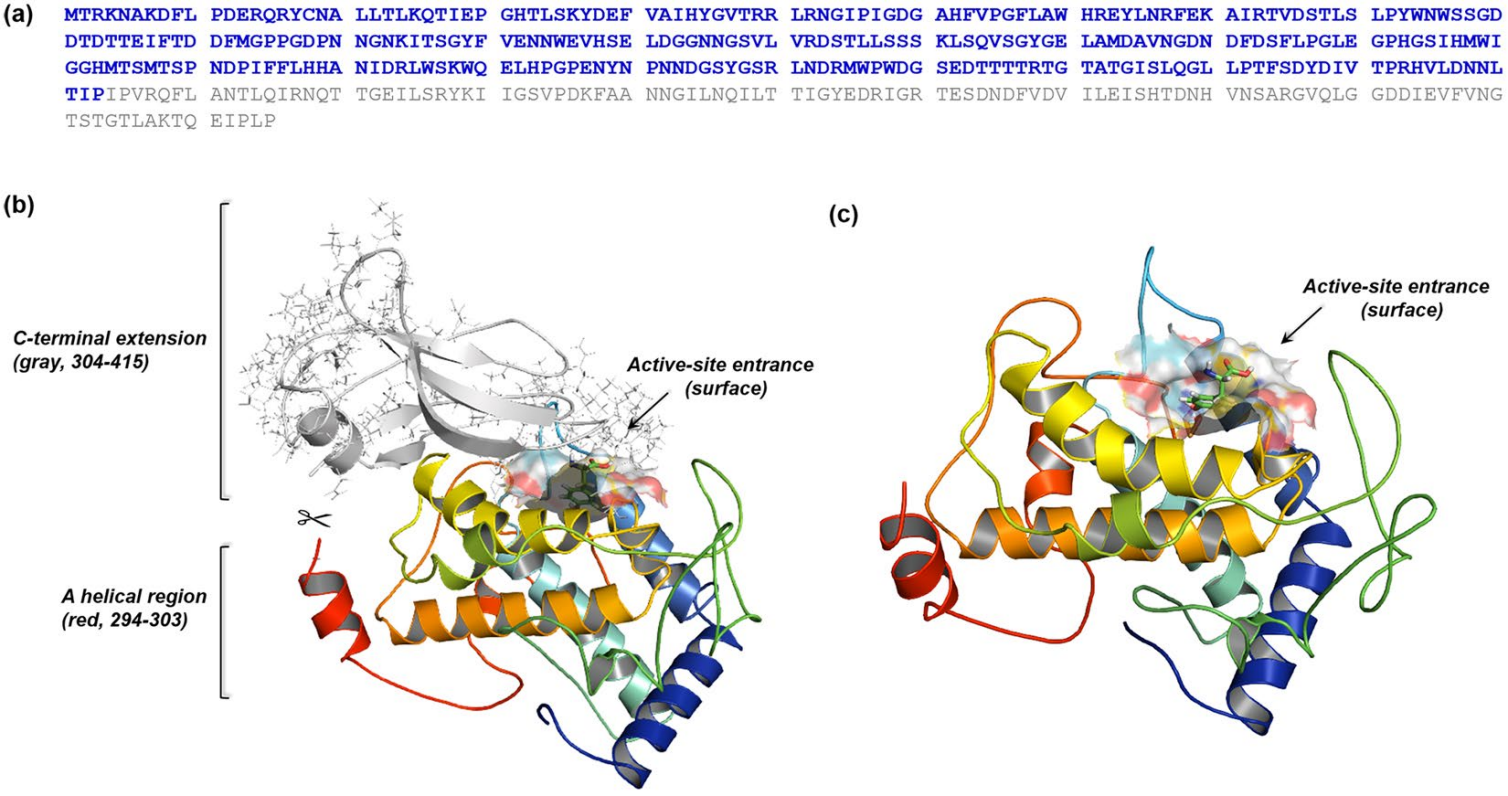
Schematic representation of mTyr-CNK. (**a**) Amino acid sequence of mTyr-CNK (blue color); the gray letters indicate the removed C-terminal extension (304–415). (**b**) Structure-based analysis of the homology model of tyrosinase-CNK. The homology model was generated by using the I-TASSER server. Gray ribbons represent the C-terminal extension (304–415) and a red ribbon structure indicates a helical region between His294 and Pro303. (**c**) Homology model of mTyr-CNK. The mTyr-CNK structure of the complex with L-tyrosine was prepared by using a CDOCKER docking simulation (color cartoon).

Recombinant tyrosinases as well as purified extracts could become attractive because natural tyrosinase extracts are not usually homogeneous (batch-to-batch variability) and are typically contaminated with other oxidizing enzymes. Moreover, recombinants could be efficiently modified in a directed manner by introducing/deleting/changing amino acid sequences at the genetic level^14–17^. We recently produced a psychrophilic tyrosinase, tyrosinase-CNK, from the marine archaeon *Candidatus Nitrosopumilus koreensis* in *E. coli* by heterologous expression^18^. Interestingly, a homology model predicted by I-TASSER revealed that tyrosinase-CNK also has a C-terminal extension (Fig. 1a,b); similar extensions have been known to prevent the activation of fungal and plant tyrosinases^18–20^. Although tyrosinase-CNK showed a high catalytic efficiency for _L_-tyrosine that was comparable to that of other known tyrosinases and phylogenetic analysis indicated that it was distinct from other known tyrosinases, we still expect that removal of the C-terminal extension in tyrosinase-CNK has great potential to significantly improve the enzyme’s catalytic property. Moreover, an engineered fragment can be more readily overproduced than the whole protein because of the whole protein’s large size and of the presence of many aggregation-prone sequences in the C-terminal extension. Producing a small fragment may also change the substrate entrance tunnel and the binding pocket of the enzyme active site, inducing different catalytic specificity and selectivity. Herein, we describe the remarkable characteristics of a mature form of tyrosinase-CNK (mTyr-CNK, 1–303) and provide strong evidence for its value in biotechnological applications as a versatile monophenol monooxygenase.

## Results and Discussion

**Figure 2:**
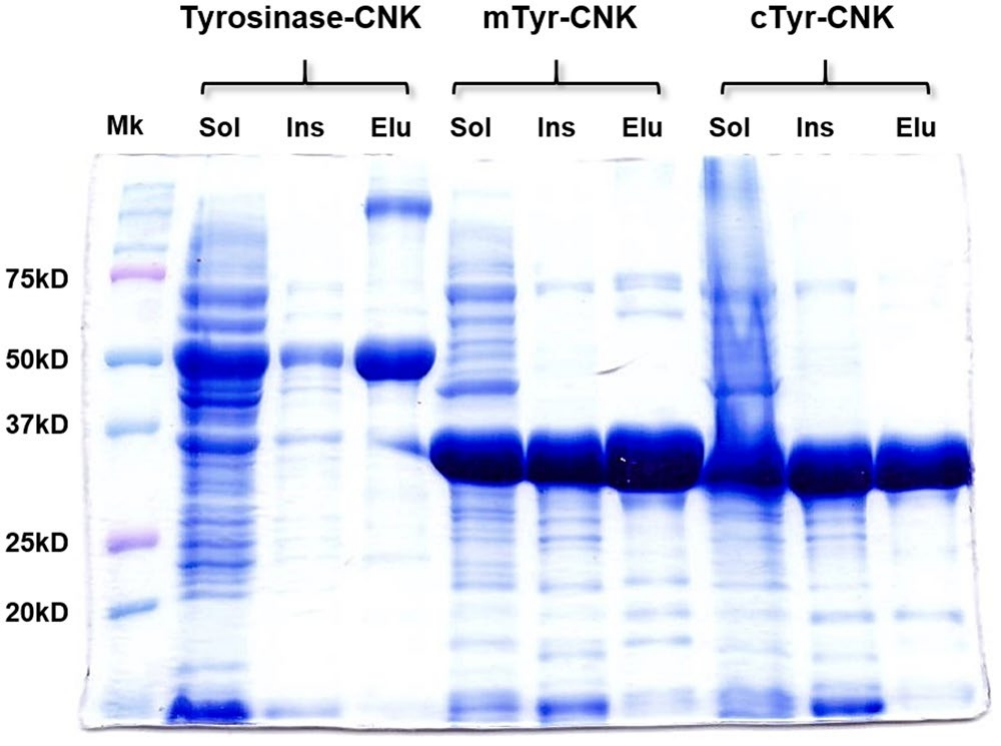
Functional expression and purification of tyrosinase-CNK, mTyr-CNK and cTyr-CNK proteins. Lanes: Sol, soluble supernatant fraction; Ins, insoluble cell debris fraction; Elu, tyrosinases purified by using Ni-NTA affinity chromatography; Mk, protein molecular weight marker.

Use of the ExPASy PeptideCutter tool (http://web.expasy.org/peptide_cutter/) revealed a potential thrombin cleavage site at Arg293; a core tyrosinase domain (cTyr-CNK, 1–293) could thus be expected to remain after proteolytic removal of C-terminal sequences (294–415). However, understanding the maturation processes of tyrosinases is still elusive, and the predicted C-terminal random-coil segment of cTyr-CNK might become destabilized and negatively influence enzyme activity^21–23^. A helical region between His294 and Pro303 was maintained, while Pro303 was likely to cause helix termination as a helix breaker^24^. Considering this likelihood, the mature form of the enzyme, mTyr-CNK, was prepared by removing the C-terminal extension (304–415), which was chosen based on a homology model that was predicted using I-TASSER (Fig. 1c)^20^. In addition, spatial aggregation propensity (SAP) analysis, which can predict aggregation-prone regions as a result of the dynamic exposure of hydrophobic patches^25^, revealed that aggregation-prone regions are mainly located in the C-terminal extension (gray line structures in 304–415) (Fig. 1b), suggesting that removal of this segment may enhance the functional folding of the remaining fragment of tyrosinase-CNK in *E. coli*. Although solubility problems vary from case to case and unexpected problems in protein folding and catalytic sites may be generally caused by introducing/deleting/changing amino acid sequences, mTyr-CNK was solubly expressed (approx. 50% of total protein, based on SDS-PAGE image analysis) in culture without chaperones at 20 °C and 200 rpm after the culture was induced with 1 mM isopropyl-β-D-thiogalactopyranoside (IPTG), despite the simultaneous formation of insoluble inclusion bodies (Fig. 2); in contrast, GroES and GroEL chaperones were required for the functional expression of tyrosinase-CNK in a previous study^18^. Finally, mTyr-CNK was simply purified by 6xHis-tag affinity chromatography to greater than 95% purity. cTyr-CNK was also solubly expressed and purified under these conditions (Fig. 2).

**Figure 3:**
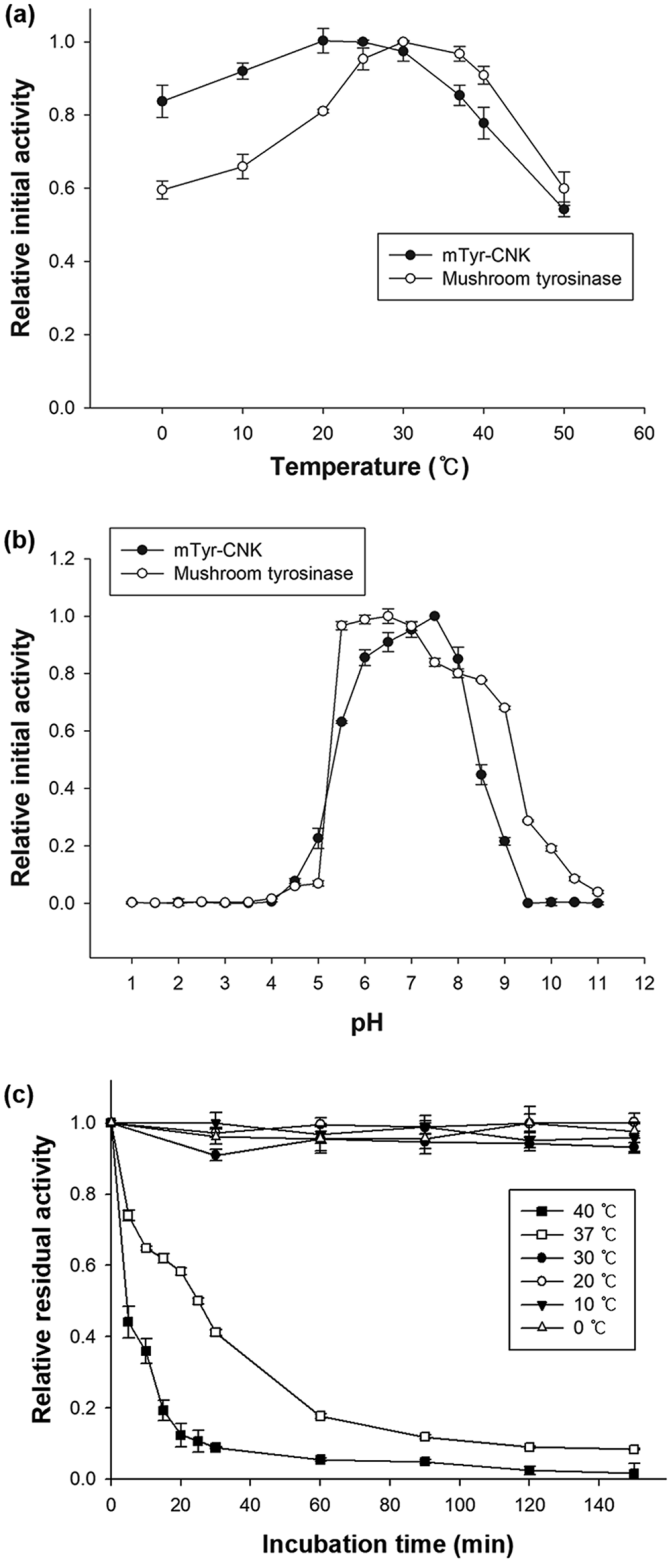
Relative initial activity of mTyr-CNK and mushroom tyrosinase at different temperatures (**a**) and pH values (**b**), and thermostability of mTyr-CNK at various temperatures (**c**).

The initial activity of mTyr-CNK against 0.05 mM _L_-DOPA substrate at room temperature and pH 6 was 1.34-fold higher than that of cTyr-CNK, as expected. Actually, cTyr-CNK had *k*_*cat*_ = 2.13 ± 0.79 (s^−1^), *K*_*m*_ = 1.60 ± 0.65 (mM), and *k*_*cat*_/*K*_*m*_ = 1.37 ± 0.07 (mM^−1^·s^−1^) for _L_-tyrosine and *k*_*cat*_ = 2.05 ± 0.10 (s^−1^), *K*_*m*_ = 0.56 ± 0.04 (mM), and *k*_*cat*_/*K*_*m*_ = 3.65 ± 0.09 (mM^−1^·s^−1^) for _L_-DOPA at pH 6 and 20 °C. cTyr-CNK exhibited lower monophenolase/diphenolase activity ratio than that of mTyr-CNK (Table 1). The removal of a helical region between His294 and Pro303 in mTyr-CNK likely changed the catalytic activity, although both mTyr-CNK and cTyr-CNK have the same core catalytic domain. Protein function is intimately linked to three-dimensional structure. The deletion of the C-terminal domain might relatively rarely affect the structural scaffold of the core catalytic domain of tyrosinase-CNK. However, this change could alter peripheral elements and affect enzymatic activity and specificity^26,27^. Copper ions should be included in the reaction buffer to effect catalytic conversion because Cu^2+^ ions can be reversibly incorporated into the active site, as observed for tyrosinase-CNK. Remarkably, mTyr-CNK was optimally active at room temperature (20–25 °C), and more than 80% of maximum catalytic activity was retained even at 0 °C because of the enzyme’s psychrophilic nature (Fig. 3a). mTyr-CNK was highly active throughout a broad range of temperature and pH values, similar to commercially available mushroom tyrosinase (Fig. 3a,b). Most impressively, mTyr-CNK exhibited the significantly high monophenolase/diphenolase activity ratio (*V*_max_ mono/ *V*_max_ di) of 3.83, and the *k*_*cat*_ */K*_*m*_ value of the monophenolase reaction was only slightly lower (87%) than that of the diphenolase reaction (Table 1), whereas the oxidation of catechols typically proceeds two orders of magnitude faster than the hydroxylation of monophenols^17,28^. The activity ratio (*V*_max_ mono/ *V*_max_ di) of mTyr-CNK was approximately two-fold higher than that of tyrosinase-CNK based on the previously reported kinetic parameters of tyrosinase-CNK: *k*_*cat*_ = 4.3 ± 1.08 (s^−1^), *K*_*m*_ = 9.2 ± 2.4 (mM), and *k*_*cat*_/*K*_*m*_ = 0.47 (mM^−1^·s^−1^) for _L_-tyrosine and *k*_*cat*_ = 2.2 ± 0.47 (s^−1^), *K*_*m*_ = 2.6 ± 0.56 (mM), and *k*_*cat*_/*K*_*m*_ = 0.85 (mM^−1^·s^−1^) for _L_-DOPA at pH 6 and 25 °C^18^. The turnover number and binding affinity of the enzyme for _L_-tyrosine were higher than those for _L_-DOPA, possibly resulting from a reorganization of the active site and the relatively high accessibility of the active site to substrates based on previously proposed structure-function correlations for other known tyrosinases^4,29^, although biochemical and structural investigations of mTyr-CNK are still required to account for its surprisingly high monophenolase/diphenolase activity ratio. Moreover, when the residual initial activity was measured after the enzyme was exposed to different temperatures for different lengths of time, the mTyr-CNK protein was very stable below 30 °C, while its half-lives at 37 and 40 °C were approximately 25 min and less than 5 min, respectively (Fig. 3c). This instability at temperatures greater than 30 °C could be utilized to efficiently and selectively inactivate the enzyme after its reaction in biotechnological applications^30^. These features are attractive in reactions in which unwanted autooxidation in catechol-related biocatalytic processes needs to be minimized and in enhancing the modification of phenolic moieties of polymeric macromolecules. Moreover, the enzyme solution is expected to retain activity well under practical enzyme storage conditions such as in refrigerators (2–8 °C) and freezers (−20 °C), based on its high storage stability below 30 °C.

**Table 1:** Kinetic parameters for mTyr-CNK at pH 6.

Temperature (°C)	Substrate	*K*_m_ (mM)	*k*_cat_ (s^−1^)	*k*_cat_/*K*_m_ (mM^−1^∙s^−1^)	*V*_max_ Mono/ *V*_max_ Di	(*k*_cat_/*K*_m_ Mono)/ (*k*_cat_/*K*_m_ Di)
20	_L_-tyrosine	2.12 ± 0.14	3.33 ± 0.18	1.57 ± 0.01	3.83	0.87
	_L_-DOPA	0.49 ± 0.04	0.87 ± 0.07	1.81 ± 0.26		
25	_L_-tyrosine	2.42 ± 0.63	3.25 ± 0.81	1.35 ± 0.01	3.78	1.02
	_L_-DOPA	0.67 ± 0.24	0.86 ± 0.25	1.33 ± 0.08

**Figure 4:**
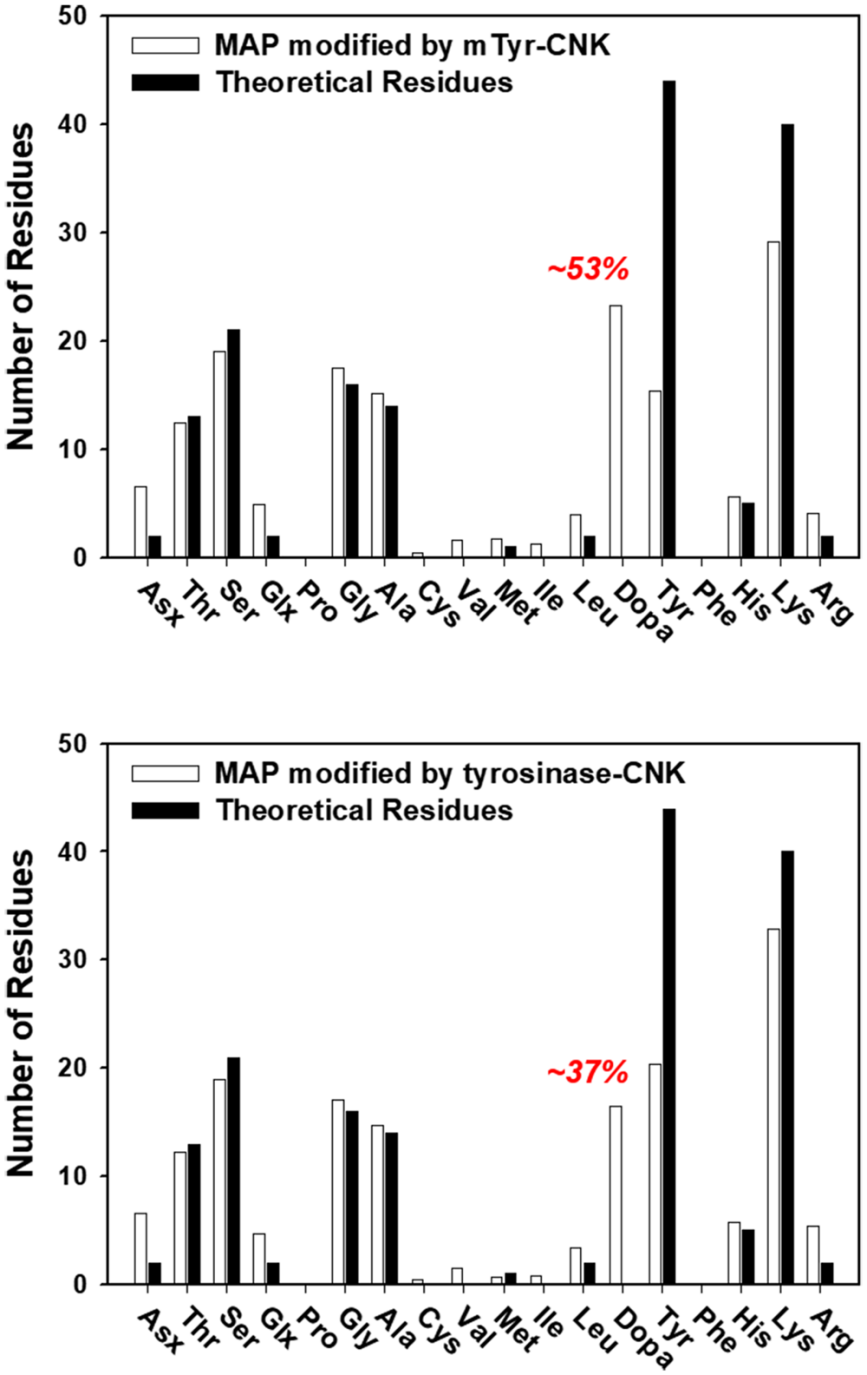
Amino acid composition analysis of MAP to determine the *in vitro* DOPA modification yield of reactions with mTyr-CNK (**a**) and tyrosinase-CNK (**b**).

As an example of these reactions, mTyr-CNK was applied to the *in vitro* DOPA modification of recombinant mussel adhesive protein. Mussel adhesive protein has been suggested to be a representative DOPA-tethered biomaterial, and the DOPA is crucial for rapid, strong underwater adhesion^31^. However, practical applications of natural mussel adhesive proteins have been greatly hampered due to the extremely low extraction yield of the natural proteins^32^. Using recombinant mussel adhesive proteins could be an alternative approach because of the ability of these techniques to produce large amounts of protein, but a low yield of *in vitro* DOPA modification (<15%) continues to be a critical issue in ensuring the high performance of the proteins as promising biomaterials^33–35^. Here, recombinant mussel adhesive protein fp-151 (referred to as MAP in this paper) was used as a model protein to study *in vitro* DOPA modification, and its DOPA content was determined by using an amino acid composition analysis. As a result, mTyr-CNK dramatically improved the modification yield (53%) over that of previously reported experiments (<15%) that had been conducted with mushroom tyrosinase (Fig. 4a). Tyrosinase-CNK also exhibited a significantly high modification yield (approximately 37%, Fig. 4b). Relatively high active-site accessibility to the tyrosine moieties of MAP as well as an enhanced monophenolase/diphenolase activity ratio under acidic conditions that could cause this significant improvement in *in vitro* DOPA modification.

In summary, a functional tyrosinase with a significantly high monophenolase/diphenolase activity ratio and enhanced catalytic efficiency against _L_-tyrosine was prepared. The enzyme, mTyr-CNK, was not only highly active at a wide range of temperatures and pH conditions and retained more than 80% of its maximum activity at 0 °C but was also very stable below 30 °C. The homology model structure suggested that the active site is relatively accessible for macromolecular phenolic substrates, even though exact experimental three-dimensional structures may provide direct evidence for the distinguished monophenolase/diphenolase activity ratio of mTyr-CNK. *In vitro* DOPA modification of MAP, as a model substrate, was efficiently achieved in high yields via biocatalysis and can be directly applied to the preparation of DOPA-tethered biomaterials for biocompatible and strong underwater adhesion. Moreover, these results showed that mTyr-CNK has a high potential for use as a monophenol monooxygenase in biomedical and industrial applications such as the biosynthesis of pharmaceutical catechol intermediates and phenolic phytochemicals, tyrosinase-based biosensors for the detection and quantification of catechol-producing compounds, and the detoxification of phenolic and substituted phenolic compounds.

## Methods

### Vector construction and strains

*Escherichia coli* DH5α (Life Technologies, Carlsbad, CA, USA) cells were used as a host for recombinant vector preparation, and *E. coli* BL21 (DE3) (Merck KGaA, Darmstadt, Germany) was used for the expression of the recombinant tyrosinases. The *E. coli* cells were grown in Luria-Bertani (LB) medium with 50 µg ampicillin mL^−1^ (Sigma-Aldrich, St. Louis, MO, USA). The target mTyr-CNK (1–303) gene was constructed from a previously optimized tyrosinase-CNK gene sequence by using a polymerase chain reaction; N-terminal *Nde*I and C-terminal *Xho*I restriction sites were added to the sequence during PCR, and the product was then introduced into a pET23b + vector (Novagen, Darmstadt, Germany), which contains a strong T7 promoter and a six-histidine (His_6_) sequence. The identity of the final vector construct (pmTyr-CNK) was confirmed by direct sequencing. The cTyr-CNK (1–293) gene was similarly inserted into the pET23b+ vector, resulting in pcTyr-CNK.

### Production and purification of the recombinant tyrosinases

Functional tyrosinase-CNK was overexpressed and purified by co-expression with chaperone proteins in *E. coli* based on our previously described method^18^. On the other hand, the co-expression strategy was not used for the production of functional mTyr-CNK and cTyr-CNK proteins. A single colony of these cells from a freshly streaked plate was grown in 50 mL of LB medium with 50 µg ampicillin mL^−1^ at 37 °C and 200 rpm until the OD_600_ of the culture was between 0.8 and 1.0. IPTG (1 mM final concentration; Sigma-Aldrich) was added to the culture to induce protein overexpression, and the cells were further incubated at 20 °C and 200 rpm for 20 h. Cells were harvested by centrifugation at 15,840 × g for 10 min at 4 °C, and the resulting pellets were stored at −80 °C. To purify the recombinant tyrosinases, we began by resuspending the cells in 5 mL of lysis buffer (50 mM NaH_2_PO_4_, 300 mM NaCl and 10 mM imidazole; pH 8.0) per gram of wet weight. The cells were disrupted by using an ultrasonic cell disrupter (ULH-700S; Ulsso High-Tech Co., Cheongwon, Korea) at 30% power for 15 min with a 3-sec on pulse and a 10-sec cooling period between each burst. The lysate was centrifuged at 15,840 × g and 4 °C for 10 min. The supernatant was collected and applied to a Ni-nitrilotriacetic acid (Ni-NTA) affinity purification column (Qiagen, Germantown, MD, USA); wash buffer (50 mM NaH_2_PO_4_, 300 mM NaCl and 20 mM imidazole; pH 8.0) and elution buffer (50 mM NaH_2_PO_4_, 300 mM NaCl, 250 mM imidazole and 0.02 mM CuSO_4_; pH 8.0) were used to isolate pure tyrosinase^18^. The expression level and purity of the proteins were monitored by sodium dodecyl sulfate-polyacrylamide gel electrophoresis (SDS-PAGE), whose gels were analyzed with image analysis software (CLIQS; TotalLab Ltd., Newcastle upon Tyne, UK). The protein concentration was assessed by using the bicinchoninic acid (BCA) assay method (Sigma-Aldrich).

### Optimum pH and temperature

To determine the optimum pH and temperature of mTyr-CNK enzyme activity, this activity was quantified at different pH by measuring the formation of _L_-dopachrome from _L_-DOPA. The assay was performed in a 200-μL volume that included 50 mM reaction buffer (glycine-HCl buffer at pH 1–3, sodium acetate buffer at pH 3.5–5, sodium phosphate buffer at pH 5.5–7, tris-HCl buffer at pH 7.5–9, and glycine-NaOH buffer at pH 9.5–11), 0.01 mM CuSO_4_, 0.05 mM _L_-DOPA and 0.4 µM purified mTyr-CNK. The formation of L-dopachrome was monitored by measuring the absorbance at 475 nm. Relative activities were similarly determined at different temperatures at a fixed pH value (pH 6). The reaction was performed in a 200-μL volume that contained 50 mM Tris buffer (pH 6.0), 0.01 mM CuSO_4_, 0.05 mM _L_-DOPA and 0.4 µM purified mTyr-CNK. Mushroom tyrosinase (Sigma-Aldrich) was also used for comparisons of temperature and pH activity profiles. All measurements were performed in triplicate in 96-well plates at room temperature and were monitored by using a multiplate reader.

### Kinetic parameters *K*_*m*_ and *k*_*cat*_

Kinetic parameters characterizing mTyr-CNK activity were determined by measuring the formation of _L_-dopachrome from _L_-tyrosine and _L_-DOPA using a spectrometric assay. The assay was performed in a 200-μL volume that contained 50 mM Tris buffer (pH 6.0) and 0.01 mM CuSO_4_. For the kinetic studies, 0.4 μM purified enzyme was used with substrate (_L_-tyrosine and _L_-DOPA) concentrations from 0.025–0.0625 mM. The formation of _L_-dopachrome was monitored by measuring the absorbance at 475 nm and converting the absorbance using a molar extinction coefficient of 3600 M^−1^ cm^−1^ ^36^. All measurements were performed in triplicate in 96-well plates and monitored using a multiplate reader with a light path of 0.582 cm.

### Thermostability of mTyr-CNK

The thermostability was based on the relative enzyme activity that remained after the enzyme had been exposed to various temperatures. The residual activities were measured at intervals and in a 200-μL volume that contained 50 mM Tris buffer (pH 6.0), 0.01 mM CuSO_4_, 0.05 mM _L_-DOPA and 0.4 µM purified mTyr-CNK. All measurements were performed in triplicate in 96-well plates at room temperature and were monitored by using a multiplate reader.

### *In vitro* DOPA modification

Lyophilized recombinant MAP fp-151 powder was dissolved to a final concentration of 1 mg/mL in a solution containing 50 mM Tris buffer (pH 6.0) and 25 mM ascorbic acid (as an antioxidant). Either purified tyrosinase-CNK or mTyr-CNK was added to a final concentration of 0.01 mg/mL. The mixture was incubated for 3 h with gentle shaking at RT, dialyzed against 10 mM acetic acid and then stored at −80 °C for further analysis.

### Amino acid composition analysis

Briefly, 25 μL of phenol was added to 500 µL of the tyrosinase-treated MAP solution in 6 M HCl. After the oxygen was removed using argon gas, acid hydrolysis was conducted by incubating the sample at 156 °C for 1 h. The protein hydrolysate was washed, and the solvent evaporated with distilled water and methanol. Then, the amino acid composition was analyzed using an ion exchange column and a ninhydrin-based detection system (Amino acid analyzer S4300; Sykam, Eresing, Germany).

### Computational analysis of mTyr-CNK

A structural homology model of mTyr-CNK was constructed using the amino acid sequence from the iterative threading assembly refinement (I-TASSER) server of the Zhang Lab (http://zhanglab.ccmb.med.umich.edu/I-TASSER), and the estimated C-score, TM-score and RMSD value were 0.21, 0.74 ± 0.11 and 5.8 ± 3.6 Å, respectively. The model structure was analyzed using Discovery Studio software (Dassault Systems BIOVIA, Discovery Studio Modeling Environment, Release 2016, San Diego: Dassault Systems, 2017). Calculations of protein ionization, residual pK values, and SAP (aggregation scores) and docking simulations (CDOCKER) were performed at pH 6.0 using Discovery Studio modules and the CHARMm force field. PyMol 1.8.6.2 (Schrodinger, LLC, Cambridge, MA, USA) was used to visualize the results.
